# Anesthetic Implications in Managing a Case of Primary Hyperaldosteronism: A Case Report

**DOI:** 10.7759/cureus.35502

**Published:** 2023-02-26

**Authors:** Renjith Ravi, Mahesh Prabhu, Baby Thampuru Vamadevan

**Affiliations:** 1 Anesthesiology, Chazhikattu Hospital, Thodupuzha, IND; 2 Anesthesiology, New Medical Center (NMC) Specialty Hospital, Abu Dhabi, ARE; 3 Anesthesiology, Burjeel Medical City, Abu Dhabi, ARE

**Keywords:** spironolactone, adrenal adenoma, conn's syndrome, hyperaldosteronism, hypokalemia

## Abstract

Primary hyperaldosteronism (also called Conn’s syndrome) is a rare condition of the adrenal glands characterized by excessive secretion of the hormone aldosterone, which regulates the balance of water and electrolytes in the body, and maintains blood volume and pressure. Hyperaldosteronism causes sodium and water retention, hypokalemia, hypertension, and muscle weakness. Common cause of primary hyperaldosteronism is an adrenal adenoma or bilateral adrenal hyperplasia. A 36-year-old female presented with hypertension, hypokalemia and muscle cramps, and on further evaluation by computed tomography (CT) scan was found to have a right adrenal adenoma. She was scheduled for a right-sided laparoscopic adrenalectomy. We report the successful peri-operative anesthetic management of this patient who had an uneventful intra-operative and post-operative course.

## Introduction

Hyperaldosteronism is characterized by excessive production of aldosterone in the body. It can be divided into primary and secondary hyperaldosteronism [1]. Primary hyperaldosteronism (Conn’s syndrome) is due to the overproduction of aldosterone from the adrenal cortex, which could be due to unilateral adrenal adenoma in a majority of the cases (60%), whereas bilateral adrenal hyperplasia is seen in 30% of cases [2]. Secondary hyperaldosteronism which occurs due to excessive activation of the renin-angiotensin-aldosterone system (RAAS) can be seen in physiological states like hypovolemia or in pathological conditions like heart failure, chronic liver disease, or renal artery stenosis. Conn’s syndrome is more commonly seen in females in the age group of 30-50 years. Patients usually present with hypertension, muscle cramps, weakness, and rarely cardiac arrhythmia secondary to hypokalemia, which may be fatal. Blood pressure can range from normotensive to severe hypertension and occasionally resistant hypertension in 20% of cases [3]. Sodium reabsorption, volume expansion, and increased peripheral vascular resistance are the causative factors for hypertension in hyperaldosteronism. Moderate-to-severe hypokalemia can cause neuromuscular symptoms such as fatigue, muscle weakness, muscle cramps, and cardiac arrhythmias. Diagnostic aids include hypokalemia, increased aldosterone levels, decreased renin levels, high plasma aldosterone concentration to plasma renin activity (PAC/PRA ratio), and imaging modalities like CT scan [4,5]. Potassium supplementation along with spironolactone and antihypertensives are the mainstay of medical management. The definitive treatment is the surgical removal of the adenoma which can present various challenges to anesthetists, especially during the surgical handling of the adrenal gland intra-operatively.

## Case presentation

**Figure 1:**
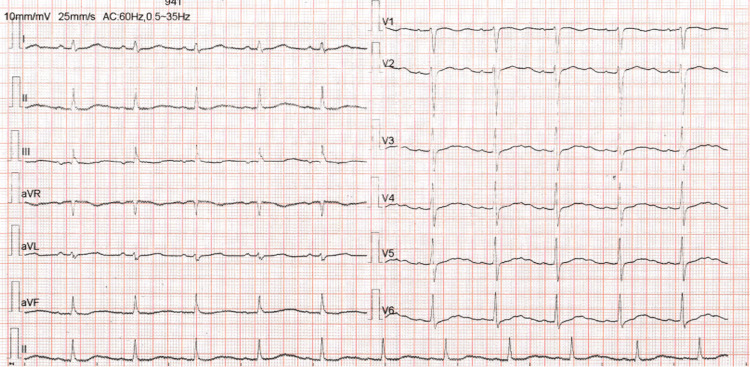
Electrocardiograph revealing prominent "U" waves in leads V2 and V3.

**Figure 2:**
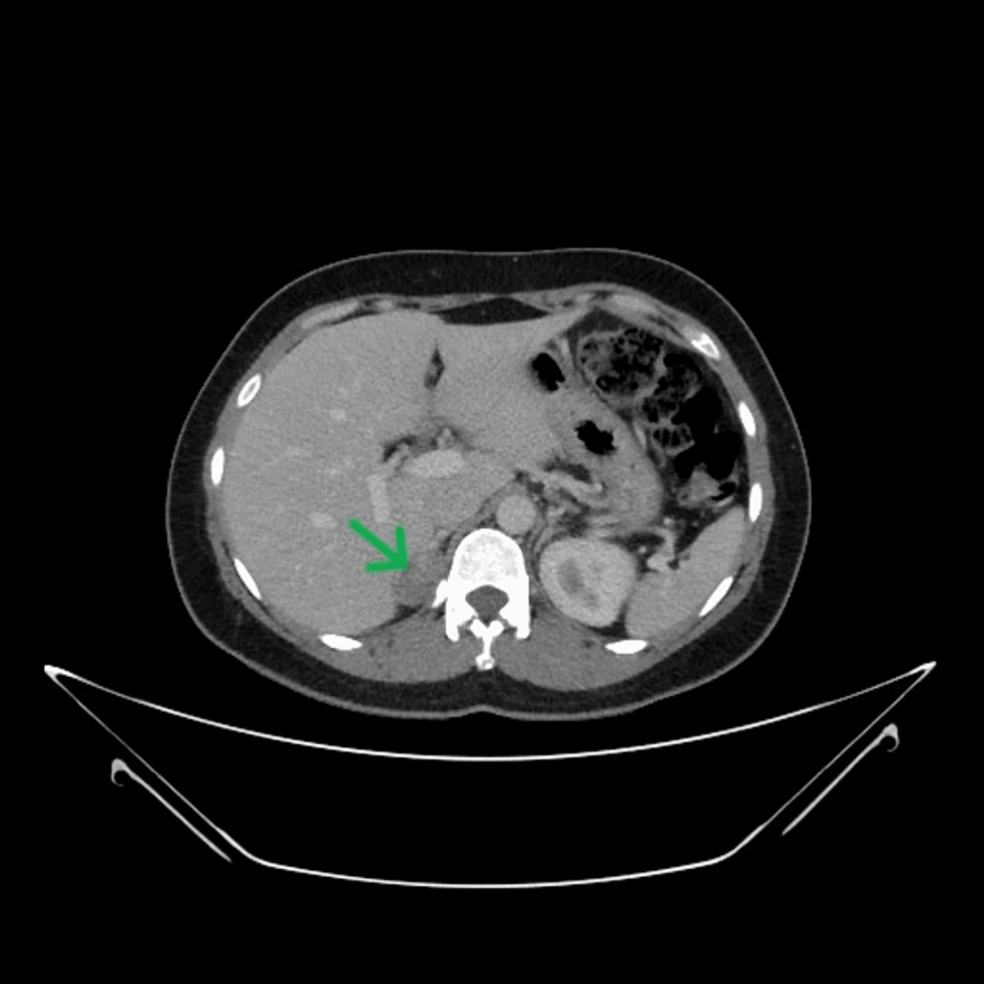
Computed tomography of the abdomen - adrenal adenoma pointed with arrow.

A 36-year-old woman with a history of hypertension presented to the outpatient clinic with muscle weakness, cramps, insomnia, and tiredness. The patient was on oral amlodipine 5 mg twice daily for the past four years for hypertension. She was evaluated in the endocrinology clinic and was found to have hypokalemia. The serum potassium was 2.82 mmol/L, while the serum sodium level was 142 mmol/L. A 24-hour urine sample showed normal metanephrine and normetanephrine levels. Further evaluation showed a low plasma renin activity, less than 0.167 ng/mL/h, and serum aldosterone value of 32 ng/dL, with a high PAC/PRA ratio of 191 (Table 1). Electrocardiograph revealed "U" waves predominantly in leads V2 and V3 (Figure 1). The CT scan of her abdomen showed a focal lobulated lesion in the right adrenal gland measuring 31.1x21 mm suggestive of an adenoma (Figure 2). All other biochemistry results and clinical assessments were within normal range. The patient was started on a potassium supplement, spironolactone 50 mg twice daily and telmisartan 40 mg once daily. She was scheduled for right-sided laparoscopic adrenalectomy after pre-operative optimization of her hypertension and potassium levels.

**Table 1:** Biochemical parameters of the patient with their normal reference range.

Blood results	Patient values	Normal values
Potassium	2.8 mmol/L	3.5-5.5 mmol/L
Sodium	142 mmol/L	135-145 mmol/L
Chloride	98.1 mmol/L	97-109 mmol/L
Bicarbonate	28.6 mmol/L	22-29 mmol/L
Plasma aldosterone concentration (PAC)	32 ng/dL	7-30 ng/dL
Plasma renin activity (PRA)	0.167 ng/mL/h	0.7-3.3 ng/mL/h
PAC/PRA	191	<30

During the preanesthesia assessment, the patient had a pulse rate of 60 beats/minute, blood pressure of 140/80 mmHg, and room air saturation of 98%. The airway and systemic examinations were within normal limits. The patient’s serum potassium had been corrected to 4.6 mmol/L by then and the serum sodium level was 136 mmol/L. Chest radiograph was normal and the echocardiogram revealed grade 1 diastolic dysfunction, with no evidence of left ventricular hypertrophy. All other blood investigations including liver and kidney function tests were within normal limits. The patient was advised to continue spironolactone and amlodipine and withhold the telmisartan on the morning of the surgery. She was explained the anesthesia techniques and risks involved for which consent for general anesthesia was obtained.

In the operating room, the patient was attached to standard anesthesia monitors including 5 lead ECG, heart rate, pulse oximeter (SpO_2_), non-invasive blood pressure (NIBP), end-tidal carbon dioxide (EtCO_2_) and train-of-four (TOF) monitor for continuous neuromuscular monitoring. She had a 20-gauge intravenous cannula in situ and was premedicated with an injection of midazolam 1 mg and an injection of fentanyl 100 mcg. Under local anesthesia, the left radial artery was cannulated for continuous blood pressure monitoring. After preoxygenation, general anesthesia was induced with propofol (2 mg/kg) in incremental doses and rocuronium (1 mg/kg). Intravenous dexamethasone 4 mg was also administered during induction. Intravenous lidocaine 1.5 mg/kg was given 90 seconds before laryngoscopy to avoid the pressor response to intubation. The patient was intubated with a 7.0 mm cuffed endotracheal tube and anesthesia was maintained with an air-oxygen mixture (1 L/min), sevoflurane targeted to an age-adjusted minimum alveolar concentration (MAC) of 0.8-1 and remifentanil infusion (0.5 mcg/kg/min). The right internal jugular vein was cannulated using a 7 French triple-lumen catheter and the patient was turned to the left lateral position on a kidney bridge. All the pressure points were adequately padded and protected. She was connected to a forced air warmer and continuous temperature monitoring was done using a nasopharyngeal probe. After creating a pneumoperitoneum, there was as a 30% fall in the mean blood pressure, which was managed with an intravenous crystalloid fluid bolus of 500 mL and two doses of ephedrine 6 mg. The patient received intermittent doses of rocuronium. Serum potassium levels were monitored twice using arterial blood samples intra-operatively, which were within normal limits. There were no significant hemodynamic fluctuations noted during the handling of the adrenal gland by the surgeon and the patient remained stable throughout the surgery. The total blood loss was 400 mL and the surgery lasted for 3 h. Toward the end of the surgery, the patient received morphine 3 mg and an intravenous paracetamol 1 g for analgesia. The patient was reversed with neostigmine 2.5 mg and glycopyrrolate 0.4 mg and extubated smoothly after achieving adequate tidal volume and muscle power (TOF ratio >0.9). She was shifted to the post-anesthesia care unit (PACU) for observation and was discharged five days later with an uneventful post-operative course.

## Discussion

Primary hyperaldosteronism, also known as Conn’s syndrome, is a rare tumor caused due to excess secretion of the mineralocorticoid hormone aldosterone from the adrenal cortex. The increased aldosterone levels in the body lead to systemic hypertension and increased plasma volume, which is due to aldosterone-induced sodium and water reabsorption in the distal renal tubular cells. Patients with Conn’s syndrome can also present with fatigue, muscle cramps, and muscle weakness, which is non-progressive and due to increased potassium loss in the urine [5]. Our patient had multiple episodes of muscle cramps and weakness before diagnosis and was treated by potassium correction in the internal medicine clinic. On her further visits, a detailed evaluation by the endocrinologist led to the diagnosis of Conn’s syndrome. Further, hypokalemia could become severe and exacerbated by diuretics, used for the management of hypertension. Normally, 98% of body potassium is located intracellularly and hence chronically low serum potassium could be associated with large intracellular deficits [6]. It has been suggested that excess aldosterone might be a risk factor for arrhythmias occurring due to left ventricular hypertrophy, cardiac fibrosis predominantly in the left atrium, or a combination of both [7]. Along with potassium, hydrogen ions are also pumped out of the renal tubular cells resulting in metabolic alkalosis. As reported by various authors, hypokalemia and hypertension and alkalosis need to be optimized before taking up these patients for surgery [8,9]. Spironolactone, a potassium-sparing diuretic, is the drug of choice and given in doses up to 400 mg/day helps in the correction of hypokalemia and alkalosis and in the restoration of normovolemia in the patient. Patients with resistant hypertension may require additional antihypertensive medications like angiotensin-converting enzyme (ACE) inhibitors or angiotensin receptor blockers (ARB) [10]. Our patient had resistant hypertension and a combination of amlodipine, spironolactone, and telmisartan was used to obtain adequate pre-operative blood pressure control. Features of end-organ damage secondary to hypertension need to be evaluated, optimized, and documented before taking up the patients for surgery.

Laparoscopic removal of the adrenal adenoma is the surgical treatment of choice as it has minimal post-operative pain, early mobilization, and recovery of the patient. Surgery can correct hyperaldosteronism in the majority of cases, although it requires around a year or more for hypertension to resolve. The best response to surgical treatment appears to be associated with the presence of an adenoma, age under 44 years, duration of hypertension of fewer than five years, and a positive pre-operative response to spironolactone [11]. Our patient fulfilled the above criteria and was an ideal candidate for surgery. Untreated hypertension secondary to primary hyperaldosteronism could lead to acute complications like stroke, myocardial infarction, and intracranial bleed [12]. Manipulation of the adrenal gland during tumor removal may cause cardiovascular instability due to the secretion of catecholamines, although this is not as severe as with catecholamine-secreting tumors of the adrenal (pheochromocytoma) [13]. A short-acting alpha-blocker like phentolamine is very useful in controlling blood pressure in such situations. In our case, there was no hypertension or tachycardia noted during the surgical manipulation of the adenoma. Hypokalemia theoretically prolongs the action of non-depolarizing neuromuscular blocking agents. It is also known to suppress baroreceptor tone and a combination of other factors like multiple antihypertensive medications including ACE inhibitors, diuretics, anesthetic drugs, positive pressure ventilation, laparoscopic insufflation, and patient position in kidney bridge can all contribute to hypovolemia, which should be treated aggressively. In our patient telmisartan was stopped on the morning of surgery and anesthesia was induced with titrated doses of propofol along with adequate co-loading of the patient with intravenous fluids during induction. However, a fall in blood pressure was noted on peritoneal insufflation in the lateral kidney bridge position which responded to crystalloid and ephedrine boluses. We used remifentanil infusion as it is an ultra-short-acting opioid which can be rapidly titrated for various levels of surgical stimuli. It provides deep analgesia and stable hemodynamics intraoperatively. The adenoma was removed successfully, and no other major hemodynamic fluctuations were noted. The patient was extubated smoothly and transported to the post-anesthesia care unit. On follow-up of the patient in the endocrinology clinic one month after surgery, the aldosterone levels had reduced to 4.6 ng/dL and patient was on a single medication of amlodipine 5 mg alone for control of hypertension, indicating the successful outcome of our surgical management.

## Conclusions

Adrenal adenomas requiring surgery need a multidisciplinary effort, including an endocrinologist, anesthesiologist, and surgeon. Conn’s syndrome presents a unique challenge to anesthesiologists in view of the associated electrolyte disturbances and hypertension-related complications. Adequate pre-operative evaluation which includes ruling out other adrenal gland tumors like pheochromocytoma, correction of electrolyte and metabolic abnormalities, appropriate anesthesia techniques, and ensuring peri-operative hemodynamics helps in successful management and positive patient outcomes.
